# Synthesis of Micro-Electrolysis Composite Materials from Blast Furnace Dust and Application into Organic Pollutant Degradation

**DOI:** 10.3390/nano12234275

**Published:** 2022-12-01

**Authors:** Xiangrong Zeng, Ting Xie, Bin Zeng, Lijinhong Huang, Xindong Li, Wanfu Huang

**Affiliations:** 1 School of Resource and Environmental Engineering, Jiangxi University of Science and Technology, Ganzhou 341000, China; 2 Jiangxi Environmental Engineering Vocational College, Ganzhou 341000, China; 3 School of Rare Earth and New Materials Engineering, Gannan University of Science and Technology, Ganzhou 341000, China; 4 School of Civil and Surveying & Mapping Engineering, Jiangxi University of Science and Technology, Ganzhou 341000, China; 5 Faculty of Science and Engineering, WA School of Mines, Minerals, Energy and Chemical Engineering, Curtin University, Perth 6152, Australia

**Keywords:** blast furnace dust, micro-electrolysis, organic pollutants, degradation

## Abstract

A micro-electrolysis material (MEM) was successfully prepared from carbothermal reduction of blast furnace dust (BFD) and coke as raw materials in a nitrogen atmosphere. The MEM prepared from BFD had strong ability in removing methyl orange, methylene blue, and rose bengal (the removal rates of methyl orange and methylene blue were close to 100%). X-ray diffraction showed that the iron mineral in BFD was ferric oxide, which was converted to zero-valent iron after being reduced by calcination. Scanning electron microscopy showed that nano-scale zero-valent iron particles were formed in the MEM. In short, the MEM prepared from BFD can effectively degrade organic pollutants.

## 1. Introduction

Blast furnace dust (BFD) is discharged with blast furnace flue gas during the iron and steel smelting process, and is collected by dry bag dust removal. The BFD has fine and uneven particle size, and is composed mainly of iron oxide and carbon, as well as harmful impurities such as lead, zinc, and alkali metal oxides [1]. This solid waste will cause serious harm to the environment [2,3]. According to statistics, the annual output of BFD in China alone will exceed 20 million tons in 2020 [2], which contains a lot of valuable metals such as zinc and iron [4]. However, since the accumulation of lead, zinc, and other alkali metals will affect blast furnace smelting, it is difficult to recover the valuable metals in BFD [5]. Therefore, BFD cannot be recycled in large quantities. In addition, due to the small particle size of BFD, it is easy to be transported by wind, causing environmental pollution and damage to the ecological environment [6]. Therefore, it is very important to study the rational utilization of BFD.

In recent years, much research has been conducted on the application of BFD [7], including the recovery of valuable metals such as zinc, iron, and lead [8,9,10]. Other applications of BFD have also been studied, including the use of blast furnace slag and glass waste to prepare foamed glass-ceramics [11], and the use as a raw material to prepare geopolymers [12], black iron oxide pigment [13], catalysts [14], and catalytic ceramic fillers [4]. Therefore, it is of great significance to develop a suitable treatment process for efficient use of BFD.

Iron-carbon micro-electrolysis developed in Europe in the 1960s is a low-cost, efficient, and environmentally-friendly method, and a promising wastewater treatment technology [15]. Its principle is to use the metal corrosion method to form a galvanic cell to treat wastewater. Iron-carbon micro-electrolysis is now used in many fields of wastewater treatment, including constructed wetland [16,17], pharmaceutical wastewater [18,19,20], petroleum wastewater, landfill leachate [21], ionic liquid wastewater [22], chemical wastewater [23,24], dye wastewater [25,26], and electroplating wastewater [27,28,29]. During the preparation of iron-carbon micro-electrolysis materials (MEMs), Fe^0^ and activated carbon are first uniformly mixed in a particular proportion and then immersed in wastewater to form a large number of microscopic primary cells [24,30]. The electrode reaction can be represented as follows [31,32,33]:

When the wastewater contacts with iron and carbon, the following electrochemical reactions occur:

anode: Fe − 2e → Fe^2+^, *E^θ^*(Fe^2+^/Fe) = −0.44V

cathode: 2H^+^ + 2e → 2[H]→H_2_, *E^θ^*(H^+^/H_2_) = 0V

When oxygen is present, the cathodic reaction is as follows:

O_2_ + 4H^+^+ 4e → 2H_2_O, *E^θ^*(O_2_/H_2_O) = +1.23V

O_2_ + 2H_2_O + 4e → 4OH^−^, *E^θ^*(O_2_/OH^−^) = +0.40V

The above reactions show that the nascent Fe^2+^ produced by the anode and the [H] generated by the cathode have high chemical activity and can effectively destroy the carbon chains of organic pollutants [34], eventually forming CO_2_, H_2_O, and inorganic ions [35]. Fe^3+^ generated by Fe oxidation is gradually hydrolyzed to Fe(OH)_3_ colloidal flocculant, which can effectively adsorb and condense pollutants in water, enhancing the purification effect of wastewater [36,37,38]. Because the Fe-C micro-electrolysis system does not require additional power, it can effectively treat organic pollutant wastewater. Therefore, the Fe-C micro-electrolysis system has attracted growing attention in the treatment of organic pollutants.

In this work, we used BFD as the iron source and coke as the carbon source to prepare MEM from carbothermic reduction. This work is aimed to determine whether the waste generated in the smelting industry can be used to prepare MEM, and to evaluate the effect of the MEM on the dye degradation. Results show that the MEM prepared from BFD can rapidly degrades azo dyes, and is a treatment process with great prospects and good treatment effect on wastewater decolorization. Then, the structure and morphology of the material were characterized by X-ray diffraction (XRD) and scanning electron microscopy (SEM), and the possible decolorization mechanism was discussed by ultraviolet spectroscopy.

## 2. Materials and Methods

### 2.1. Materials

The BFD used here came from a local steel smelter with an iron grade of 35.18%. The reducing agent was coking coal with a fixed carbon content of 85.32%. The coal was crushed to the size below 0.15 mm, then bagged and sealed for storage. All chemicals were of analytical grade and purchased from China National Pharmaceutical Group. The tubular furnace was purchased from Hefei Kejing Material Technology Co., Ltd. (OFT-1200X-S, Hefei, China). Deionized water was used in the experiments.

### 2.2. Preparation and Experiment of Materials

The method for preparing micro-electrolysis composites from BFD was as follows: BFD (50 g) was mixed with coke (25 wt%), sodium carboxymethyl (5 wt%), and deionized water (20 wt%), and then manually made into 10 mm diameter pellets. The pellets were put into a tubular electric furnace under a nitrogen atmosphere (0.1 L/min) of calcination. The heating rate of a tubular furnace was 8 °C/min. After calcination, the reduced pellets were cooled to room temperature, mixed, and sealed for storage.

**Figure 1:**
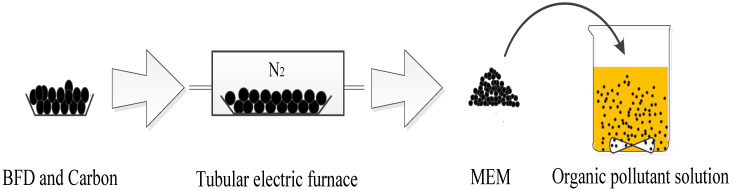
Diagram of micro-electrolysis process.

The experiments were carried out in 500 mL beakers each with a 400 mL solution of 100 mg/L organic pollutants. A certain amount of MEM was added to each beaker, and the decolorization reaction was carried out at a stirring speed of 500 rpm. All experiments were carried out in a magnetic stirrer, and the temperature was controlled by a water bath. After decolorization for the preselected time, the sample wastewater obtained from the syringe was filtered through a 0.45 μm aqueous membrane filter for analysis. All experiments were repeated three times. The flow chart of micro electrolysis is shown in Figure 1.

### 2.3. Analysis and Characterization

The samples were characterized by an SEM meter (Quanta FEG 250, FEI, USA) and an XRD device (SMART APEXII, Bruker, Germany). An ultraviolet-visible spectrophotometer (UV-1800, Mapada, China) was used to analyze the residual concentrations of the target pollutants in the solutions. The detection wavelengths of methyl orange, methylene blue, and rose bengal were 464, 664, and 545 nm, respectively. The high-concentration sample solutions were diluted to a certain concentration and then measured. The decolorization efficiency γ of target pollutants is defined as follows:

(1) $$\gamma =\frac{C_{0}-C_{t}}{C_{0}}\times 100\%$$

where $C_{0}$ and $C_{t}$ are the initial concentration and the concentration at time *t* (min) of the target pollutant, respectively.

## 3. Results and Discussion

### 3.1. Effects of Preparation Conditions on Organic Pollutant Degradation

The key influence factors on the formation of ZVI from iron minerals during the MEM preparation from BFD include calcination temperature, mass ratio of Fe/C, and calcination time. The formation of ZVI in turn affects the organic pollutant degradation performance of MEM. To improve the performance of MEM, we studied the effects of preparation conditions on methyl orange decolorization efficiency.

**Figure 2:**
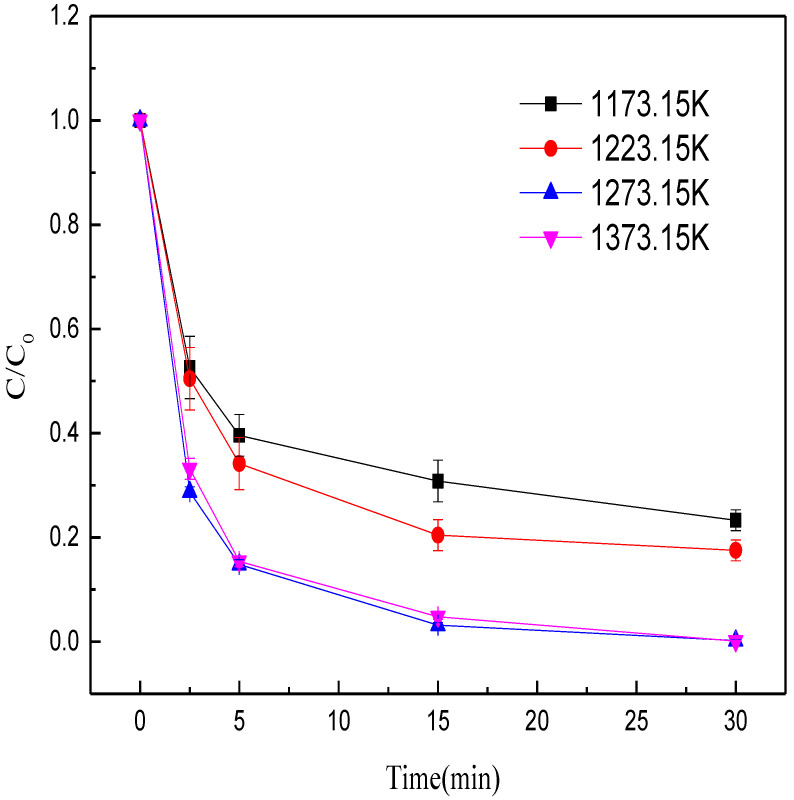
Effects of roasting temperature on the decolorization efficiency of methyl orange.

First, the effects of calcination temperature (1173.15, 1223.15, 1273.15, 1373.15 K) on methyl orange decolorization were studied. Other conditions were kept constant: calcination time of 40 min, mass ratio of Fe/C of 4:1, initial pH at 6.3, dose of MEM at 10 g/L, and solution temperature at 28 °C. When the calcination temperature rose from 1173.15 to 1273.15 K, the methyl orange decolorization efficiency by MEM significantly increased within 15 min from 69.17% to 96.83% (Figure 2). However, when the calcination temperature rose to 1373.15 K, the methyl orange decolorization efficiency within 15 min was 95.20%. The possible reason was that with the temperature rise at early stage of calcination, the iron minerals in the BFD were gradually reduced to ZVI, which was favorable for the decolorization of methyl orange. The iron minerals were fully reduced to ZVI at the calcination temperature of 1273.15 K. As the calcination temperature further rose to 1373.15 K, the ZVI particles aggregated [39], which decreased the chance of contact between ZVI and methyl orange, thus decreasing the efficiency of methyl orange removal. Hence, in the subsequent experiments, the optimal calcination temperature was set at 1273.15 K. 

**Figure 3:**
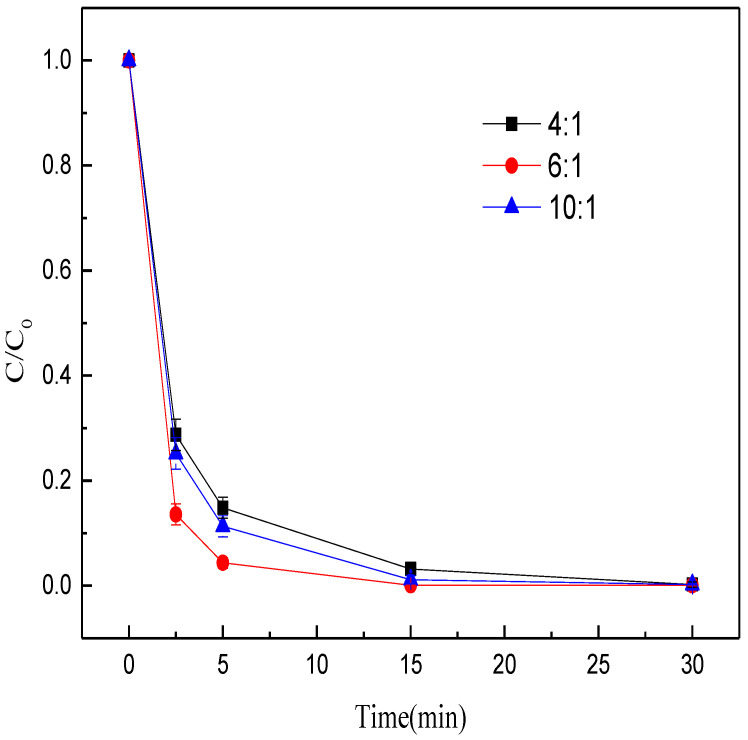
Effects of mass ratio of Fe/C on the decolorization efficiency of methyl orange.

To investigate the effects of coal amount on methyl orange decolorization, we conducted batch experiments with different mass ratios of Fe/C (4:1, 6:1, 10:1) while keeping other parameters constant. Clearly, the methyl orange decolorization efficiency first increased and then declined with the rise of mass ratio of Fe/C (Figure 3). As the mass ratio of Fe/C rose from 4:1 to 6:1, the methyl orange decolorization rate within 15 min increased from 96.84% to 99.90%. When the mass ratio of Fe/C further increased to 10:1, the decolorization efficiency dropped to 98.85%. The possible reason was that when the mass ratio of Fe/C was insufficient, the iron minerals cannot be fully reduced to ZVI. However, when the mass ratio of Fe/C was excessive, the coal was reserved in MEM, which decreased the decolorization rate of methyl orange. Hence, in the subsequent experiments, the optimal mass ratio of Fe/C was set at 6:1. 

**Figure 4:**
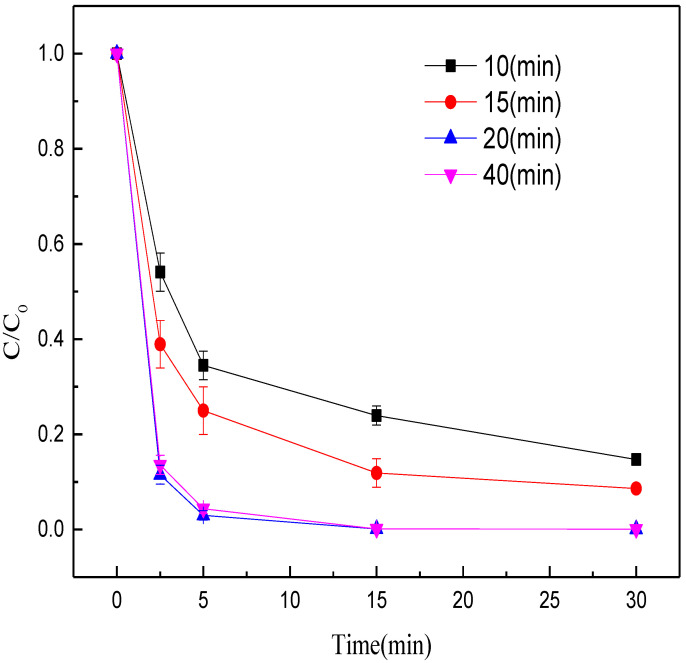
Effects of calcination time on the decolorization efficiency of methyl orange.

Then, the effects of calcination time (10, 15, 20, 40 min) on the methyl orange decolorization were evaluated at 1000 °C and with the Fe/C mass ratio of 6:1 while other conditions were unchanged. The decolorization efficiency increased with the prolonging of calcination time from 10 to 20 min (Figure 4). When the calcination time exceeded 20 min, the decolorization efficiency did not improve. The possible reason is that appropriate prolonging of calcination time is favorable for the production of ZVI. After the iron minerals were fully reduced, prolonging the calcination time did not increase the ZVI content, but led to ZVI aggregation instead, which weakened the decolorization efficiency of methyl orange. Thus, the optimal calcination time was set at 20 min. 

Based on the above data, we determined the optimal conditions of MEM preparation to be calcination temperature at 1000 °C, Fe/C mass ratio of 6:1 and calcination time of 20 min. Then the MEM prepared under the optimum conditions was used in the subsequent experiments.

### 3.2. Effects of Reaction Parameters on Organic Pollutant Degradation

Micro-electrolysis reaction parameters such as dose of MEM, aqueous pH, and type of pollutants all affect the organic pollutant degradation in the micro-electrolysis system. Then, the MEM prepared under the optimum conditions was used to study these parameters.

**Figure 5:**
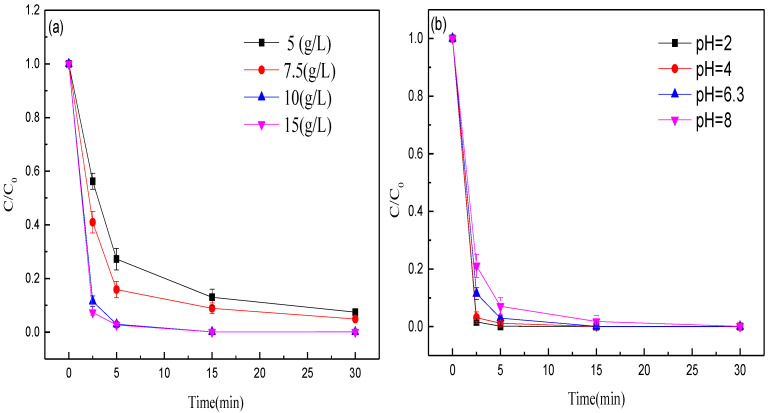
Effects of (**a**) dose of MEM, and (**b**) aqueous pH on the decolorization efficiency of methyl orange.

First, the effect of the dose of MEM on methyl orange decolorization efficiency was explored. The methyl orange decolorization efficiency was improved with the increase of MEM dosage (Figure 5a). The methyl orange decolorization rates within 5 min with the doses of 5, 7.5, 10 and 15 g/L MEM were 72.78%, 84.13%, 97.04%, and 97.31%, respectively. As the reaction time was prolonged to 30 min, the methyl orange decolorization rate was 92.55%, 95.09%, 99.93%, and 99.96%, respectively. An increase in the dose of MEM offers extra surfactant sites, thus quickening the degradation reaction and improving the decolorization efficiency. Thus, the optimal dose of MEM was 10 g/L.

Then, the effects of pH (within 2.0–8.0) on methyl orange decolorization were studied. The methyl orange decolorization efficiency was weakened with the rise of initial pH (Figure 5b). The decolorization rates within 2.5 min at pH 2.00, 4.00, 6.28 and 8.00 were 98.34%, 96.87%, 88.5%, and 78.87%, respectively. Clearly, the methyl orange decolorization effect of MEM is better under acid conditions. The possible reason is that under acid conditions, iron is more easily oxidized to Fe^2+^, so the positively-charged surface of Fe^2+^ and the negatively-charged dye molecules facilitate the adsorption of dyes onto the material surface. 

**Figure 6:**
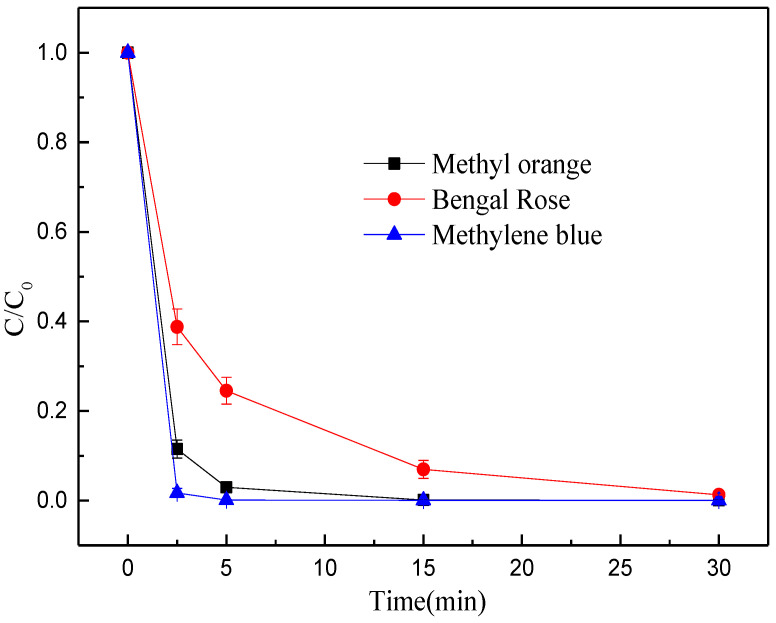
Decolorization efficiency of MEM organic pollutants.

Besides, the performances of MEM were evaluated by detecting some commonly-detected organic pollutants in degraded wastewater, including methylene blue, eosin Y and acidic magenta. The MEM prepared from BFD well degraded methyl orange, methylene blue, and rose bengal (Figure 6). Especially, the treatment efficiency on methylene blue was the best, as the decolorization efficiency was up to 99% within 5 min and was close to 100% within 15 min. The treatment efficiency on methyl orange was only second to that of methylene blue, and the decolorization rate was 97% within 5 min and was close to 100% after 30 min. The decolorization rate of rose bengal within 30 min was 99%. Based on the above experimental results, the MEM prepared from BFD is a promising for degradation of organic pollutants.

### 3.3. Characterization and Mechanism of Organic Removal

**Figure 7:**
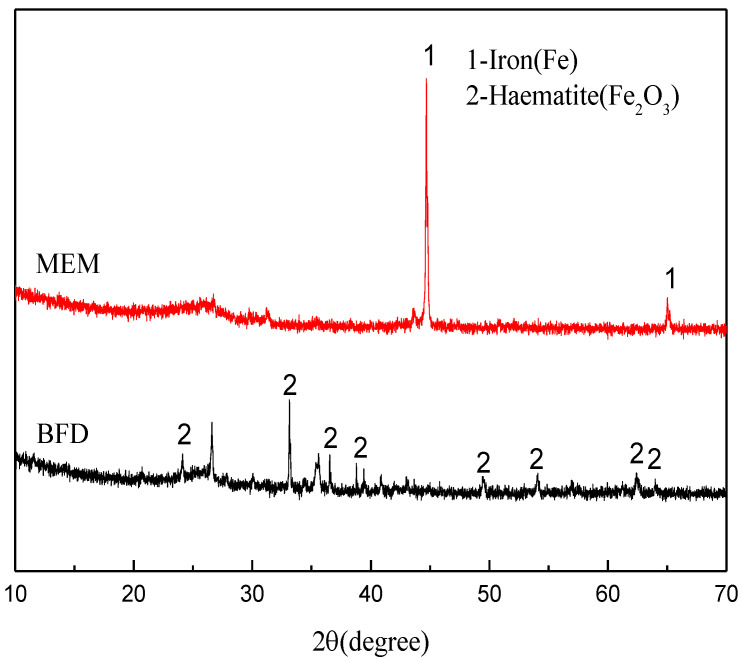
XRD patterns: BFD; Micro electrolytic composite material prepared at 1273.15 K.

Figure 7 shows the XRD patterns of BFD and the micro-electrolysis system. BFD mainly shows the peaks of Fe_2_O_3_, but has no diffraction peak of ZVI (Figure 7). After calcination at 1000 °C, the diffraction peaks of Fe_2_O_3_ disappeared, while the peaks of ZVI appeared, indicating the iron in BFD was basically converted to ZVI after the calcination at 1000 °C. 

**Figure 8:**
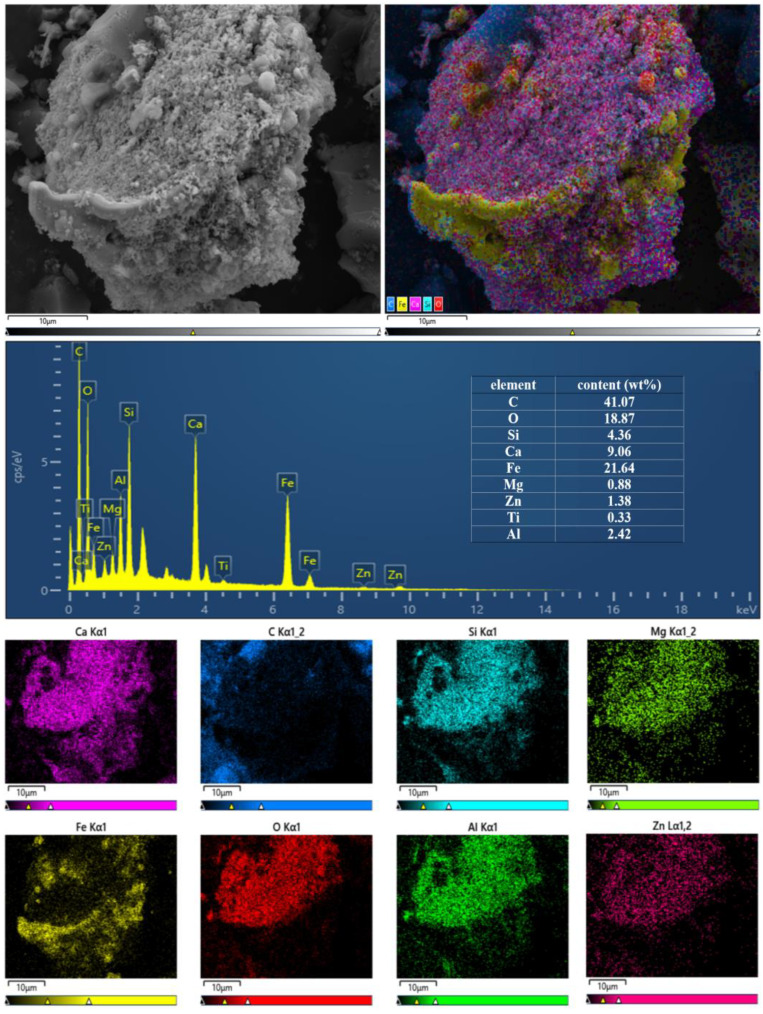
EDS and SEM of the micro electrolytic composite material prepared at 1273.15 K.

Figure 8 shows the EDS and SEM images of MEM after 20 min of calcination at 1000 °C in the nitrogen atmosphere with the Fe/C mass ratio of 6:1. EDS and SEM showed abundant nanometer and micrometer ZVI particles were formed in the MEM and adhered to the carbon surface, increasing the contact probability with pollutants, and quickening the pollutant treatment rate. EDS also showed the MEM contained abundant iron and carbon and had other substances including Ca, Si, Mg and Al. 

**Figure 9:**
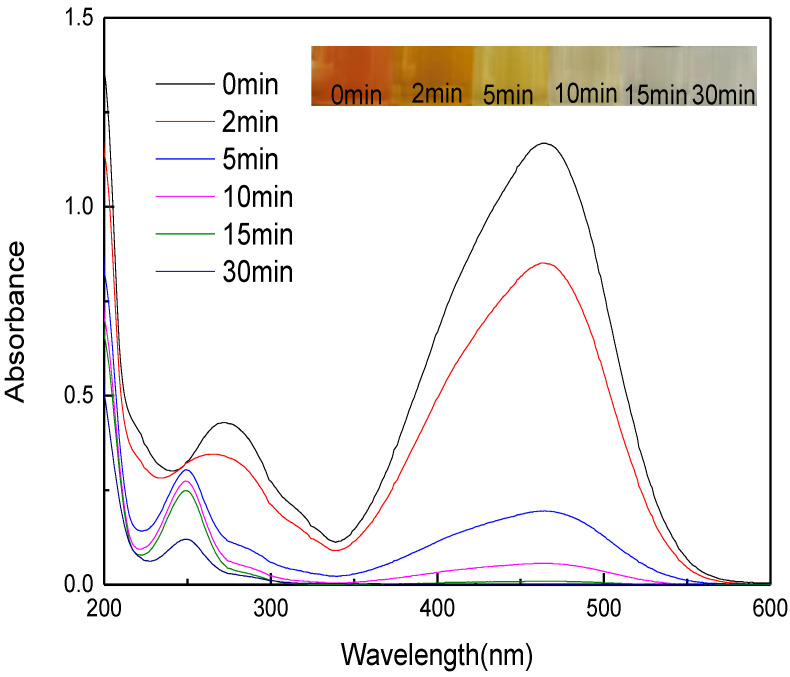
UV-VIS spectra of methyl orange during degradation by MEM (reaction temperature of 28 °C).

Figure 9 displays the UV-VIS spectral variation during methyl orange degradation. The strongest peak of methyl orange at 465 nm is ascribed to the conjugated structure formed from the azo bond. As the reaction proceeded, the peak at 272 nm was significantly weakened, and the peak at 465 nm gradually disappeared, suggesting the azo bond was slowly destroyed. After 5 min of reaction, a new peak at 248 nm appeared, which may be caused by the intermediate sulfonic acid (SA) [40], indicating micro-electrolysis can degrade methyl orange by destroying the azo bond [39,41]. After 15 min of reaction, the peak at 465 nm basically disappeared, and the peak at 248 nm was significantly weakened in intensity, as methyl orange turned from orange to be colorless at this moment. 

## 4. Conclusions

MEMs were successfully prepared from carbothermal reduction of BFD and coke in a nitrogen atmosphere. XRD and SEM showed the iron mineral in BFD was Fe_2_O_3_, and the abundant nanometer and micrometer ZVI particles formed after calcination reduction adhered to the carbon surface. EDS also showed the MEM contained abundant iron and carbon and had other substances including Ca, Si, Mg, and Al. The formation of ZVI particles increased the probability of contact with pollutants and accelerated the pollutant treatment rate. The MEM prepared from BFD well degraded methyl orange, methylene blue, and rose bengal. The decolorization rates of methylene blue and methyl orange after 30 min of reaction were close to 100%, and the decolorization rate of rose bengal was 99%.

## Data Availability

Not applicable.
